# Direct and indirect effects of adiposity on markers of autonomic nervous system activity in older adults

**DOI:** 10.1371/journal.pone.0303117

**Published:** 2024-05-16

**Authors:** Michael S. Jarrett, Travis Anderson, Laurie Wideman, Paul G. Davis

**Affiliations:** 1 Department of Kinesiology, University of North Carolina at Greensboro, Greensboro, North Carolina, United States of America; 2 Department of Exercise Physiology, Winston Salem State University, Winston Salem, North Carolina, United States of America; 3 United States Olympic and Paralympic Committee, Colorado Springs, Colorado, United States of America; Muhimbili University of Health and Allied Sciences School of Medicine, UNITED REPUBLIC OF TANZANIA

## Abstract

Several cardiovascular disease (CVD) risk factors (e.g., hypertension, poor glycemic control) can affect and be affected by autonomic nervous system (ANS) activity. Since excess adiposity can influence CVD development through its effect on hypertension and diabetes mellitus, it is important to determine how adiposity and altered ANS activity are related. The present study employed structural equation modeling to investigate the relation between adiposity and ANS activity both directly and indirectly through biological variables typically associated with glycemic impairment and cardiac stress in older adults. Utilizing the Atherosclerosis Risk in Communities (ARIC) dataset, 1,145 non-smoking adults (74±4.8 yrs, 62.8% female) free from known CVD, hypertension, and diabetes and not currently taking beta-blockers were evaluated for fasting blood glucose (FBG), insulin, and Hb_A1c_ concentrations, waist circumference (WC), blood pressure (BP), and markers of ANS activity. WC was recorded just above the iliac crest and was used to reflect central adiposity. Resting 2-minute electrocardiograph recordings, pulse wave velocity, and ankle-brachial index data were used to assess the root mean square of successive differences in RR intervals (RMSSD) and the pre-ejection period (PEP), markers of parasympathetic and sympathetic activity, respectively. FBG, insulin, and Hb_A1c_ inferred a latent variable termed glycemic impairment (GI), whereas heart rate and diastolic BP inferred a latent variable termed cardiac stress (CS). The structural equation model fit was acceptable [root mean square error of approximation = 0.050 (90% CI = .036, .066), comparative fit index = .970, Tucker Lewis Index = 0.929], with adiposity having both significant direct (β = 0.208, p = 0.018) and indirect (β = -.217, p = .041) effects on PEP through GI. Adiposity displayed no significant direct effect on RMSSD. CS displayed a significant pathway (β = -0.524, p = 0.035) on RMSSD, but the indirect effect of WC on RMSSD through CS did not reach statistical significance (β = -0.094, p = 0.137). These results suggest that adiposity’s relation to ANS activity is multifaceted, as increased central adiposity had opposing direct and indirect effects on markers of sympathetic activity in this population of older adults.

## Introduction

Major risk factors for cardiovascular disease (CVD) include both lifestyle choices (e.g., physical inactivity, poor diet) and physiological conditions (e.g. diabetes mellitus, hypertension, obesity) [1]. These physiological conditions have been associated with altered autonomic nervous system (ANS) activity [2–6]. Observed ANS activity reflects a balance between the sympathetic and parasympathetic branches, which oppose one another but are flexible and dynamic in nature. The sympathetic branch provides an overarching accelerating stimulus, promoting metabolic substrate mobilization and utilization in addition to increasing heart rate and cardiac contractile force [7]. In contrast, the parasympathetic branch is the overarching decelerator of the body, promoting substrate storage and decreased heart rate [8]. Bodily functions are regulated by the balance between these opposing systems and any prolonged disturbance or asymmetry in activity can result in the development of several clinical and physiological conditions [9,10].

ANS activity is often evaluated indirectly through indices of heart rate variability (HRV). HRV, or the time variance among intervals of consecutive cardiac cycles, is a widely used and accepted means of assessing ANS activity at the cardiac level [11]. Irregular ANS activity assessed via HRV has been heavily evaluated for its association with CVD and mortality. Specifically, chronically reduced parasympathetic and elevated sympathetic activities are associated with premature mortality, myocardial infarction, and development of type II diabetes mellitus and hypertension [12–14]. Various time and frequency domain HRV metrics can be used to estimate the level of parasympathetic output at the cardiac level [15]. The root mean square of successive differences between normal (non-ectopic) beats (RMSSD) is the primary time domain metric used to reflect vagally mediated changes, with an increase in RMSSD reflecting an increase in parasympathetic activity [16].

Some HRV metrics have been suggested to estimate sympathetic activity (namely the low frequency spectral power component and the ratio of low frequency to high frequency spectral power components), but these values are inherently limited in evaluating sympathetic output given that they are also influenced by parasympathetic activity [17]. An alternative and more independent method of evaluating sympathetic activity at the cardiac level is to monitor systolic time intervals, i.e., the amount of time between electrical stimulation of the heart and its mechanical response. Specifically, the segment of time known as the pre-ejection period (PEP), an estimate of the electromechanical delay of the left ventricle [18], can be used to accurately estimate sympathetic activity [19]. PEP has been shown to have a pronounced inverse relation with sympathetic activity in that sympathetic neural blockade stimulates an increase in PEP length, while parasympathetic blockade results in no change in PEP [20]. When used concurrently, PEP and HRV measurements can provide holistic details of ANS activity at the cardiac level.

**Fig 1:**
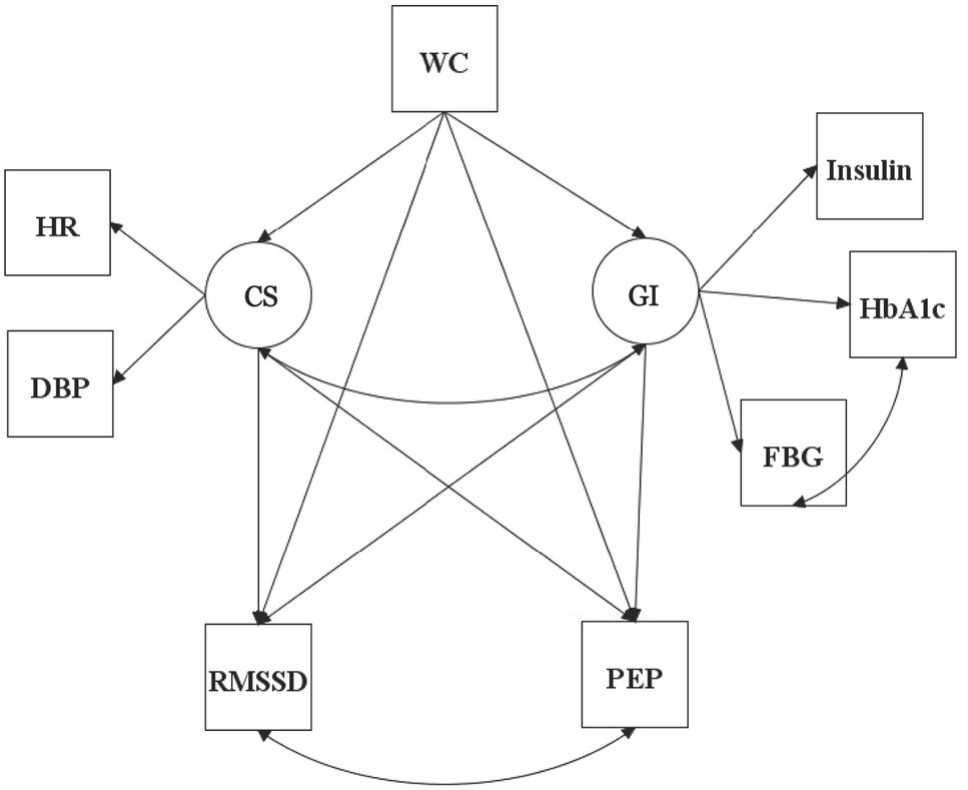
Proposed model. *A priori* model including observed variables (squares) depicting the proposed pathways for the direct and indirect effects of waist circumference on markers of ANS activity. Construction of the latent variables (circles) Cardiac Stress (CS) and Glycemic Impairment (GI) are depicted. WC, Waist Circumference; HR, Heart Rate; DBP, Diastolic Blood Pressure; Hb_A1c_, Glycosylated Hemoglobin; FBG, Fasting Blood Glucose; RMSSD, Root Mean Square of Successive Differences between normal-to-normal beats; PEP, Pre-Ejection Period.

While increased adiposity is an independent risk factor for CVD development [21], it is also a well-established risk factor for CVD comorbidities such as diabetes mellitus [22] and hypertension [23]. While significant associations between metrics of increased adiposity and altered ANS activity have been reported [24–26], it is unclear if increased adiposity has both a direct and indirect relation to ANS activity. However, evaluating the influence of adiposity on ANS activity through its risk for diabetes mellitus and hypertension development is a complex undertaking, as there is no single metric that can accurately reflect diabetic or hypertensive risk. Latent variable modeling constructs an unobserved variable that reflects the combined influence of multiple measured variables. Applying this methodology to reflect relations within our conceptual model (Fig 1) provides a more comprehensive assessment of the influences of adiposity on ANS activity. In this model, adiposity is reflected by waist circumference (WC) and has direct effects on RMSSD and PEP (markers of parasympathetic and sympathetic nervous system activities, respectively). Our model also suggests that adiposity has indirect effects on ANS activity through latent constructs representing glycemic impairment (GI) and cardiac stress (CS). We therefore aimed to more accurately model the complex associations between adiposity and ANS function, by fitting a model that included possible indirect effects through CS and GI.

## Methods

### Study population

This secondary data analysis study was reviewed (IRB #:19–0119) by the Institutional Review Board at the University of North Carolina, Greensboro on September 27th 2018 and deemed ‘NHSR’ (Not Human Subjects Research), since the data analysis did not include identifiable data. The data used in the present study was obtained from the Atherosclerosis Risk in Communities (ARIC) study. Access to the data was granted by the Biologic Specimen and Data Repository Information Coordinating Center on October 17^th^, 2018 and the current study did not have access to information that could identify individual participants during or after data collection. The ARIC dataset consists of 15,792 participants recruited from four different field centers throughout the United States of America (Minneapolis, Minnesota; Washington County, Maryland; Forsyth County, North Carolina; and Jackson, Mississippi). These participants were given a comprehensive physical examination at baseline between the years of 1987–1989 and were reevaluated every three years. Detailed methods about data collection and study design for ARIC have been published [27]. Data for the present study was derived from a fifth cycle of visits that was conducted with 5,900 participants between 2011 and 2013. For the analyses outlined here, individuals with known cardiovascular disease, those being pharmaceutically treated for hypertension or diabetes mellitus, anyone with a systolic blood pressure ≥140 mmHg and/or diastolic blood pressure ≥90 mmHg, those who identified as a smoker or that reported smoking in the last 6 months, and those taking beta-blockers at the time of evaluation were excluded. The resulting participant pool (n = 1145) was 61% female and 39% male, aged 74.6 ± 4.8 years. The majority (89%) of subjects self-identified as “white” with 11% self-identifying as “black”. Further sample characteristics are provided in Table 1.

**Table 1:** Characteristics of study population.

	Mean	Standard Deviation	Range (min, max)
N = 1,145			
Age (years)	74.6	4.8	67, 88
Mass (kg)	73.4	15.1	37.7, 145.2
Height (cm)	165.6	9.5	141, 195
Body Mass Index (kg·m^-2^)	26.67	4.60	15.89, 57.43
Waist Circumference (cm)	96.6	12.5	59, 155
Females (N = 696)	94.1	13.1	59,155
Males (N = 449)	100.4	10.4	73, 137
FBG (mg^.^dL^-1^)	104.79	15.85	65.01, 185.03
Insulin (μU·mL^-1^)	10.86	7.97	0.20, 70.02
Hb_A1c_ (%)	5.66	0.48	4.2, 8.6
DBP (mmHg)	66.4	9.3	40, 89
HR (bpm)	66	9	41, 110
RMSSD (msec)	23.52	23.14	1.22, 166.98
PEP (msec)	94.7	18.6	4.0, 173.5

### Study variables

The ARIC study defined the level of the WC measurement at just above the uppermost lateral border of the right ilium [28]. This mertic served as the sole marker for adiposity in the present study. Body mass index and percentage of body fat as determined by bioelectrical impedance analysis were provided in the ARIC dataset and were considered during model construction. In the subset of participants meeting the inclusion criteria of the current study, WC had stronger associations with other outcomes of interest compared to either body mass index or perecentage of body fat, thus, the final models utilized only WC. The construction of a latent variable for adiposity was also considered but the additional model parameters resulted in a significantly weaker model compared to the proposed model outlined in Fig 1. Thus, WC was determined to be the most appropriate metric available to serve as a marker for adiposity. Further details on the implication of this decision are outlined in the discussion section. Latent variables were constructed based on the proposed model and potential markers available from the ARIC dataset that aligned with each latent variable.

#### Glycemic impairment (latent variable)

Multiple biomarkers and indices can be used to provide a holistic evaluation of an individual’s glucose handling capacity. Thus, the manifest variables of fasting blood glucose (FBG), glycosylated hemoglobin (Hb_A1c_), and insulin were used to construct a “Glycemic Impairment” (GI) latent variable. FBG concentration provides a short-term approximation of glucose tolerance (∼8–12 hours), while Hb_A1c_ concentrations provide a marker of long-term glucose homeostasis (∼3–4 months). Furthermore, the inclusion of concurrent insulin concentration provides information on possible insulin resistance.

#### Cardiac stress (latent variable)

Two resting cardiac parameters were used to reflect the functional status of the cardiac system. The heart’s overall function is reflected through heart rate (HR) and contractility. HR is regulated by a multitude of factors and is closely related to ANS output. Previous research with the ARIC dataset showed diastolic blood pressure (DBP) to have greater associations with HRV metrics in normotensive subjects than did systolic blood pressure [29]. Additionally, DBP has a greater influence on mean arterial pressure at rest. Given both these factors, DBP appears to be a valid metric for this model. Thus, the observed variables of HR and DBP were used to construct a “Cardiac Stress” (CS) latent variable.

#### ANS activity markers (observed variables)

Resting electrocardiogram, pulse wave velocity, and ankle-brachial index data were collected in a supine position following at least eight hours of fasting. A 2-minute electrocardiogram recording was used to calculate short term HRV metrics. The root mean square of successive differences in RR intervals (RMSSD) was employed to reflect parasympathetic nervous system activity [16,26,30]. The pre-ejection period (PEP), calculated from data collected from ankle-brachial index and pulse wave velocity measurements (Omron BP-203RPEIII, Omron Healthcare co. Ltd, Kyoto, Japan), was used to indicate sympathetic nervous system activity [15,20,31].

### Model construction

Central adiposity, more than overall adiposity, is strongly related to the development of cardiometabolic diseases [32]. As such, the present model explored the effects of waist circumference (WC) on RMSSD (parasympathetic) and PEP (sympathetic), both directly and through the latent variables GI and CS. Given the inherent nature of FBG and Hb_A1c_ and their high correlation (r = 0.61, p < .001), the model allowed for a covariance between the two variables. Similarly, the latent variables of GI and CS were allowed to covary, given their strong correlation (r = 0.78, p < .001) and their shared relation to CVD through similar metabolic pathways [33]. Since structural equation model calculations, by default,assume that outcome variables in a latent variable analysis have a shared covariance, RMSSD and PEP were allowed to covary in the present analysis, even though their correlation was not statistically significant (Table 2).

**Table 2:** Pearson’s correlation coefficients of observed variables.

	FBG	Hb_A1c_	Insulin	DBP	HR	PEP	RMSSD
Hb_A1c_	0.61**	‐‐					
Insulin	0.31**	0.19**	‐‐				
DBP	0.06	0.01	0.11**	**‐‐**			
HR	0.13**	0.14**	0.18**	0.11**	‐‐		
PEP	-0.04	0.02	-0.02	0.07*	0.05	‐‐	
RMSSD	-0.05	-0.03	0.00	-0.05	-0.21**	-0.02	‐‐
WC	0.28**	0.19**	0.49**	0.18**	0.05	0.05	0.06*

Note: *p < .05

**p < .001.

### Statistical analysis

Means, standard deviations, and Pearson’s correlation coefficients for the observed variables were calculated using SPSS Statistics Version 28. The structural equation model was gauged for goodness of fit using multiple fit indices: the root mean square error of approximation (RMSEA), comparative fit index (CFI: criterion value of 0.95), Tucker Lewis index (TLI: criterion value of 0.95), and the standardized root mean square residual (SRMR: criterion value of 0.08) [34]. The direct and indirect relations outlined by the proposed model were conducted in Mplus Version 8.7 with statistical significance set at α = 0.05.

## Results

### Descriptive statistics

Anthropometric and cardiometabolic characteristics are provided in Table 1. The mean waist circumference (WC) for the total cohort was 96.6 cm, with the average for females (94.1 cm) and males (100.4 cm) indicating borderline risk of CVD. The mean values of Glycemic Impairment (GI) metrics (FBG, insulin, and Hb_A1c_) were lower than clinical cutoffs for diabetes mellitus, although the mean FBG of 104.79 mg^.^dL^-1^ is within the pre-diabetes range. Mean values for metrics of Cardiac Stress (CS; HR and DBP) were within normal ranges.

Pearson’s correlation coefficients of all observed variables are provided in Table 2. Relations among GI variables were fair to moderate (r = 0.19–0.61, all p<0.001), while the CS variables, HR and DBP were weakly but significantly related (r = 0.11, p<0.001). Waist circumference (WC) displayed fair relations (r = 0.28–0.49, all p<0.001) with all GI metrics andwas weakly but significantly related to DBP (r = 0.18, p<0.001). WC was weakly but significantly related to RMSSD (r = 0.06) and was not significantly related to PEP.

### Model analyses

The structural equation model displayed an adequate fit for the sample population with significant goodness of fit indices (RMSEA = 0.050 90% CI [0.036–0.066], SRMR = 0.028, CFI = 0.970, TLI = 0.929). Both latent variables (GI and CS) displayed significant factor loadings with their indicator variables (Table 3), suggesting a robust latent construct. Direct variance and covariance parameter estimates for the entire model are listed in Table 4.

**Table 3:** Latent variable parameter estimates.

	Parameter	Unstandardized	SE	Standardized
Glycemic Impairment (GI)	FBG	1.000	-	0.431**
	Insulin	14.964	1.418	0.711**
	Hb_A1c_	0.360	0.036	0.285**
Cardiac Stress (CS)	HR	1.000	-	0.519**
	DBP	0.385	0.100	0.213**

**Table 4:** Variance and covariance estimates.

	Parameter	Unstandardized	SE	Standardized
RMSSD	GI	17.605	13.228	0.288
	CS	-2.352	1.117	-0.524*
	WC	-0.091	0.215	-0.049
PEP	GI	-15.573	7.252	-0.318*
	CS	0.874	0.510	0.243
	WC	0.308	0.131	0.208*
Glycemic Impairment (GI)	WC	0.021	0.002	0.682**
Cardiac Stress (CS)	WC	0.074	0.022	0.178**
Covariances	FBG∼∼ Hb_A1c_	0.206	0.013	0.566**
	GI∼∼CS	0.796	0.141	0.566**
	RMSSD∼∼PEP	22.510	22.700	0.060

Note: SE, standard error; *p < .05

**p < .001.

#### Direct effects

The direct, indirect, and total effects of WC on RMSSD and PEP are provided in Table 5. WC failed to have a significant direct effect on RMSSD but had a significant direct effect on PEP (*β* = 0.208, p = .018), with a one standard deviation increase in WC being related to a 0.208 standard deviation increase in PEP.

**Table 5:** Standardized parameter estimates of direct, indirect, and total effects of waist circumference on RMSSD and PEP.

	Path	Estimate	S.E.	P-value
RMSSD				
	Direct	-0.049	0.108	0.647
	Indirect: WC›GI	0.196	0.147	0.181
	Indirect: WC›CS	-0.094	0.063	0.137
	Total Indirect	0.103	0.105	0.329
	Total	0.053	0.029	0.070
PEP				
	Direct	0.208	0.088	0.018*
	Indirect: WC›GI	-0.217	0.106	0.041*
	Indirect: WC›CS	0.043	0.034	0.205
	Total Indirect	-0.173	0.083	0.038*
	Total	0.034	0.030	0.247

*Statistically significant at *α* = 0.05.

#### Indirect and total effects

In this model, WC failed to have a significant indirect effect on RMSSD through the latent variables of GI or CS, resulting in the lack of a significant total indirect effect (the sum of all indirect effects on the dependent variable). The total effect (direct effect + total indirect effect) of WC on RMSSD (p = 0.070) and the indirect effect of WC on PEP through CS (p = 0.205) were not statistically significant. The indirect effect of WC through GI had a significant effect on PEP in that a one standard deviation increase in WC was associated with a 0.217 standard deviation decrease in PEP. Furthermore, the total indirect effect of WC on PEP was significant with a one standard deviation increase in WC being related to a 0.173 standard deviation decrease in PEP. However, the total effect of WC on PEP was not significant.

#### Other significant pathways

**Fig 2:**
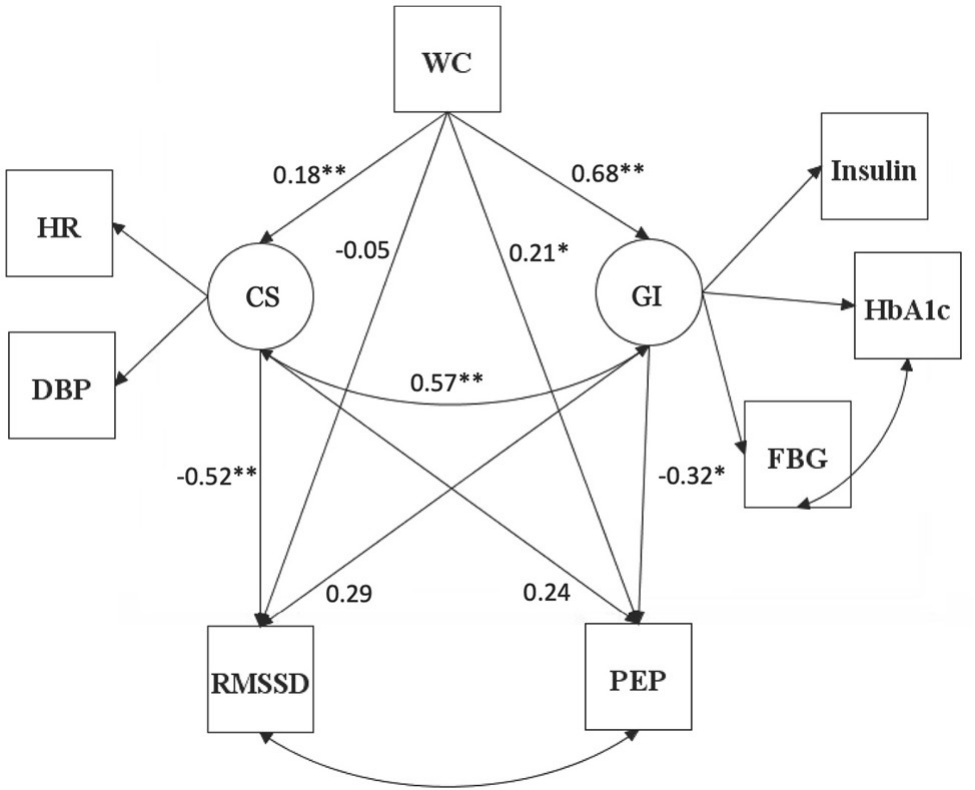
Structual equation model. Structual equation model including the standardized solutions for the direct and indirect effects of waist circumference on markers of RMSSD and PEP. Cardiac Stress, CS; Glycemic Impairment, GI; WC, Waist Circumference; HR, Heart Rate; DBP, Diastolic Blood Pressure; Hb_A1c_, Glycosylated Hemoglobin; FBG, Fasting Blood Glucose; RMSSD, Root Mean Square of Successive Differences between normal-to-normal beats; PEP, Pre-Ejection Period. Note: SE, standard error; *p < .05; **p < .001.

WC had significant effects on GI and CS with a one standard deviation increase in WC being associated with 0.682 and 0.178 standard deviation increases in GI and CS, respectively. CS had a significant effect on RMSSD in that a one standard deviation increase in CS would elicit a 0.524 standard deviation decrease in RMSSD. However, CS failed to have a significant effect on PEP. GI had a significant effect on PEP with a one standard deviation increase in GI being associated with a 0.318 standard deviation decrease in PEP. GI did not have a significant effect on RMSSD. A comprehensive depiction of the structural equation model with standardized factor loadings is provided in Fig 2.

## Discussion

The results of this study support the concept that central adiposity, as measured by WC, has significant direct and indirect effects on markers of ANS activity. Specifically, WC displayed robust associations with markers of sympathetic nervous system activity, while having little influence on parasympathetic nervous system activity when accounting for the impact of glycemic impairment markers and indices of cardiac stress. This highlights the need to directly evaluate both branches of the autonomic nervous system when discussing the relation of ANS activity and adiposity. Additionally, there is evidence that the impact of central adiposity goes beyond direct effects and that nuanced mechanisms may influence ANS function.

In this model, WC failed to have a significant direct, indirect, or total effect on RMSSD. These findings differ from earlier studies that have demonstrated direct relations between adiposity and metrics of parasympathetic activity. Windham et al. (2012) found that an increase in WC was significantly associated with a decrease in RMSSD and SDNN in middle-aged (∼45 years old), but not older (∼85 years old), participants when controlling for age, sex, race, hypertension, glucose tolerance, and physical activity. The age difference between the Windham et al. younger cohort and the present study could be a contributing factor for this difference in statistical magnitude as it has been shown that the age-related decrease in time domain metrics of HRV subsides after 60 years of age in apparently healthy individuals [35].

Our results displayed a significant direct effect of WC on PEP, suggesting a decrease in sympathetic activity with a higher WC. However, the total effect of WC on PEP was neutralized through the negative influence of GI. This model suggests that increases in central adiposity (WC) might have a blunting effect on sympathetic activity (direct effect) while simultaneously increasing sympathetic drive by increasing (worsening) indices of glycemic impairment (indirect effect). These opposing actions mitigate observable physiological changes in PEP, as reflected by the lack of a significant total effect of WC on PEP.

The direct effect of WC on PEP, suggesting a decrease in sympathetic activity with an increase in WC, seems counterintuitive, but is biologically plausible. Aging is often accompanied by an increase in sympathetic nervous system activity [36], particularly due to an impaired ability of baroreflexes to buffer changes in blood pressure [37]. Esler et al suggest that sympathetic activity at the level of the heart is normal or lower in obese individuals compared to normal weight individuals in elderly populations with hypertension [38]. The suggested concept of the obesity paradox lends credence to the notion that central adiposity could decrease sympathetic activity in elderly poulations such as the one used in the currect study. While those diagnosed with overt hypertension under older criteria (blood pressure ≥140/90 mmHg and/or under pharmaceutical treatment) were excluded from the current study, this observation of lower and/or normal sympathetic activity in obese individuals with normal to slightly eleveated blood pressure coincides with the observed significant direct effect and the general lack of a total effect of WC on PEP.

The observed indirect effect of increased WC being related to increased sympathetic activity (decrease in PEP) via an increase in GI is commonly observed in recent studies evaluating sympathetic activity and various markers of GI [39–41]. The latent variable GI reflects the combined influence of FBG, Hb_A1c_, and insulin, with any increase in these metrics resulting in increases in the GI variable. Insulin had the highest factor loading for the GI construct and is likely the primary driver for the observed effects. Elevated serum insulin concentrations are postulated as one of the major pathways linking increased adiposity (specifically central adiposity) to sympathetic nervous system overactivity [32]. In addition, direct exposure of cardiac tissue to insulin can induce positive inotropic effects through calcium dependent and independent mechanisms [42]. The present study also showed FBG and Hb_A1c_ to have significant factor loadings for the GI construct. Previous research has demonstrated increases in FBG [43–45] and Hb_A1c_ [46–48] with increases in markers of sympathetic activity. Furthermore, ANS activity reflective of cardiac autonomic neuropathy complications have been associated with diabetes mellitus [39]. Linkage between adiposity, as determined by WC, and autonomic function has been observed in groups stratify by glucose tolerance, with a higher prevalence of cardiovascular autonomic neuropathy in those with worsening glucose tolerance [49]. Similar findings were observed using dicotomus groupings based on metabolic syndrome status, with an increased prevalence of cardiovascular autonomic neuropathy in those with metabolic syndrome compared to those without [49]. These findings were independent of worsening cardiac parameters, as makers of endothelial function were similar across groups regardless of stratification method. Given this, the authors postulated that glucose tolerance was the most impactful driving force for autonomic function deterioration [49]. This concept is in agreement with the current study results, since WC only displayed a significant indirect relation with autonomic function via the latent varible GI.

Body fat distribution has been suggested to be a more potent risk factor for the development of cardiovascular disease and hypertension compared to markers of general adiposity [50]. One of the more recently explored pathways is the potential role of visceral adiposity on hypertension development via increased sympathetic nervous system activity [50]. As such, markers for visceral fat (WC) should display a more significant relation with markers of sympathic activity (PEP). However, such relations were not exhibited in the current study as shown by the insignificant indirect effects of WC on PEP through the CS construct.

Given that HRV metrics have been shown to be altered with diabetic status and impaired glycemic control, those previously diagnosed with diabetes mellitus were excluded from the current study. However, the sample population used in this secondary data analysis had a mean fasting blood glucose of 104.79±15.85 mg^.^dL^-1^, suggesting that a large portion of the group (n = 561, approximately 49% of the sample population) would be classified as pre-diabetic. In addition, there is increasing evidence that people with pre-diabetes may demonstrate signs of cardiac autonomic neuropathy [51]. Similarly, blood pressure just below the hypertensive cutoff is associated with autonomic dysfunction [4]. While the mean diastolic blood pressure of the sample population did not classify as hypertensive (66.4±9.3 mmHg), there were 102 participants (approximately 9% of the sample population) that had a DBP of ≥80 mmHg. As such, those at risk for, but not formally diagnosed with, hypertension were included in the sample. Therefore, despite the exclusion of those diagnosed with diabetes and/or hypertension, the ranges of glycemic and cardiovascular parameters in the present data set are broad enough for the investigation of their influence on autonomic function. Given that the only significant in-direct effect of WC on autonomic function was through the latent variable of GI, it is possible that the increased prevalence of elevated glycemic control markers in this cohort (compared to the prevalence of elevated blood pressure) influenced our results.

The choice of WC as our marker of adiposity over other population-based metrics (e.g., body mass index) could have influenced the observed results. Compared to body mass index, WC better reflects the amount of visceral fat [52]. Since visceral fat is more metabolically deleterious, it is a greater predictor of mortality than subcutaneous fat [53]. However, the interrelations between WC, visceral fat, and markers of parasympathetic neural activity are less straightforward. Increases in visceral fat deposits as measured by computed axial tomography (an even more accurate method of visceral fat determination) have been shown to have significant inverse relations with RMSSD in the absence of a significant correlation between WC and RMSSD, despite WC and visceral fat area being positively related [54]. Still, studies with larger cohorts (n = 8,538) suggest that WC has a stronger inverse relation to HRV metrics, specifically RMSSD, than body mass index or waist-to-hip ratio in apparently healthy populations [55]. These previously observed decreases in markers of parasympathetic activity, including RMSSD, were attributed to an increase in sympathetic activity due to an increase in adiposity, which the current study observed in the significant indirect effects of WC on PEP via GI. As a whole, the results of our study suggest that a greater association of sympathetic tone with centralized adiposity occurs without direct alterations to parasympathetic activity.

The ARIC study utilized data collected from pulse wave velocity and ankle-brachial index measurements to calculate PEP. Studies have used both methodologies independently to provide various markers of CVD risk [56,57] and monitor disease progression [58]. Additionally, these methods have been shown to provide valid approximations of systolic time intervals in the absence of more precise methods such as echocardiogram [59], giving credence to the accuracy and validity of the PEP measurements used for the current study. HRV results were derived from a 2-minute ECG rhythm strip which, depending on the metric used, could have a sizable impact on results. Considered the gold-standard of clinical HRV assessment, a recording length of 24 hours provides the most detailed evaluation of ANS activity [17]. However, shorter recordings of approximately five minutes have been successfully used in numerous studies [30]. Frequency domain measures are preferred since they are influenced to a lesser extent by shorter recording lengths than most time domain measures [11], but only time domain metrics were available in the ARIC dataset. Future studies utilizing more robust HRV metrics and longer ECG recording lengths have the potential to shed more light on the proposed relations of the study variables. Our results are strengthened by the inclusion of validated indices of both branches of the ANS. This provides an accurate, holistic representation of ANS activity without relying on the inferences from a single branch, as is a common practice when solely using HRV results. Additionally, our cohort included both male and female participants. Although sex is a potential confounder, HRV differences between the sexes has been shown to be reduced after approximately 60 years of age [60]. The current study did not evaluated sex differences in the modeled relations due to the smaller number of male subjects in the investigated cohort (n = 446). Such a sample size was insufficient to run such a complex model with this number of parameters. Thus, an accurate sex comparison of the full model could not be made in the current study. Evaluation of sex differences should be conducted in future studies utilizing an adequat sample size of both male and female participants.

The current study is not without its limitations. The cross-sectional design of our study diminishes inferences of causality in the observed relations and the directions of the effects beyond those suggested in the model development. Secondary analysis of the ARIC dataset provided a sample size that was large enough to adequately test our proposed structural equation model. However, we view this as an initial step and future longitudinal investigation is necessary to confirm any causal effects underlying the direct and indirect relations identified in the present study. In addition, while numerous adiposity markers were considered for these analyses (WC, BMI, % fat from BIA), the best model fits were found with WC. However, we acknowledge that none of the adiposity markers available in the dataset are considered gold standard assessments. The use of dual-energy x-ray absorptiometry to assess body composition and/or the use of computed axial tomography to more accurately quantify visceral fat could strengthen the observed effects in this model and explain more of the variance in ANS activity.

Interestingly, similar studies have found that adding markers of cardiovascular fitness could improve model fit parameters and be a stronger determiner of HRV indices of ANS activity [61]. The addition of cardiovascular fitness could have impactful effects on the proposed model. Specifically, an increase in cardiovascular fitness will have considerable effects on adiposity, glycemic management, and cardiac stress parameters, all of which can influence ANS activity. The current model did not account for cardiovascular fitness or physical activity status even though both have direct effects on all the study’s variables, since these variables were available for only small subsets of the ARIC cohort. While the ARIC study recorded self-reports of physical activity as well as conducted clinical measures of physical fitness (grip strength, walking tests, etc.), restricting the current study cohort to include only those that completed these measurments resulted in significant reductions in the sample size. The sample size reductions, coupled with the increased number of model parameters (i.e., adding any of the available physical fitness metrics), resulted in an inability to render a testable structural equation model. Future investigations into how cardiovascular fitness may mitigate or abolish the observed effects of adiposity on ANS activity are warranted.

In summary, our results provide support for a theoretical and statistically sound model for describing the relation of adiposity and ANS activity while accounting for the interactions of contributing physiological parameters. In a population of older, apparently healthy adults, increased central adiposity displayed opposing direct and indirect effects on markers of sympathetic activity, resulting in a lack of significant overall differences in PEP, regardless of adiposity (WC). Moreover, WC presented no significant direct or indirect effects on an index of parasympathetic activity. These results suggest that the true nature of adiposity’s relation to ANS activity is multifaceted. Use of latent structural equation modeling to uncover the complex interrelations of multiple risk factors may better illustrate the biological control systems behind cardiometabolic risk factors and CVD. This approach may more closely mirror multifactored physiological conditions such as metabolic syndrome, which is strongly influenced by the interplay of glycemic regualtion, cardiovascular health, central adiposity, and autonomic function. If it is true that adiposity directly and indirectly effects sympathetic activity, as reflected in the current model by PEP, then reducing adiposity or limiting adiposity gain during aging may promote improved autonomic function by means of both direct influence and indirect influence via alterations to glycemic regulation and cardiovascular health. This utilization of a multipronged approach is in agreement with the current clinical recommendations on how toreduce the risk of developing metabolic syndrome and CVD. Prospective interventional studies employing multiple time points should consider the current proposed and evidenced model to confirm these outcomes.

## References

[1] Tzoulaki I, Elliott P, Kontis V, Ezzati M. Worldwide Exposures to Cardiovascular Risk Factors and Associated Health Effects: Current Knowledge and Data Gaps. Circulation. 2016;133: 2314–2333. doi: 10.1161/CIRCULATIONAHA.115.008718 27267538

[2] Alam I, Lewis MJ, Morgan J, Baxter J. Linear and nonlinear characteristics of heart rate time series in obesity and during weight-reduction surgery. Physiol Meas. 2009;30: 541–557. doi: 10.1088/0967-3334/30/7/002 19458410

[3] Barutcu I, Esen AM, Kaya D, Turkmen M, Karakaya O, Melek M, et al. Cigarette Smoking and Heart Rate Variability: Dynamic Influence of Parasympathetic and Sympathetic Maneuvers. Ann Noninvasive Electrocardiol. 2005;10: 324–329. doi: 10.1111/j.1542-474X.2005.00636.x 16029383 PMC6932108

[4] Erdogan D, Gonul E, Icli A, Yucel H, Arslan A, Akcay S, et al. Effects of normal blood pressure, prehypertension, and hypertension on autonomic nervous system function. Int J Cardiol. 2011;151: 50–53. doi: 10.1016/j.ijcard.2010.04.079 20472314

[5] Soares-Miranda L, Sattelmair J, Chaves P, Duncan GE, Siscovick DS, Stein PK, et al. Physical Activity and Heart Rate Variability in Older Adults: The Cardiovascular Health Study. Circulation. 2014;129: 2100–2110. doi: 10.1161/CIRCULATIONAHA.113.005361 24799513 PMC4038662

[6] Tarvainen MP, Laitinen TP, Lipponen JA, Cornforth DJ, Jelinek HF. Cardiac Autonomic Dysfunction in Type 2 Diabetes â€”Effect of Hyperglycemia and Disease Duration. Front Endocrinol. 2014;5. doi: 10.3389/fendo.2014.00130 25152747 PMC4126058

[7] Gordan R, Gwathmey JK, Xie L-H. Autonomic and endocrine control of cardiovascular function. World J Cardiol. 2015;7: 204. doi: 10.4330/wjc.v7.i4.204 25914789 PMC4404375

[8] Thayer JF, Yamamoto SS, Brosschot JF. The relationship of autonomic imbalance, heart rate variability and cardiovascular disease risk factors. Int J Cardiol. 2010;141: 122–131. doi: 10.1016/j.ijcard.2009.09.543 19910061

[9] Pagani M, Malfatto G, Pierini S, Casati R, Masu AM, Poli M, et al. Spectral analysis of heart rate variability in the assessment of autonomic diabetic neuropathy. J Auton Nerv Syst. 1988;23: 143–153. doi: 10.1016/0165-1838(88)90078-1 3049759

[10] Zhou Y, Xie G, Wang J, Yang S. Cardiovascular Risk Factors Significantly Correlate With Autonomic Nervous System Activity in Children. Can J Cardiol. 2012;28: 477–482. doi: 10.1016/j.cjca.2012.02.003 22560462

[11] Malik M, Bigger JT, Camm AJ, Kleiger RE, Malliani A, Moss AJ, et al. Heart rate variability: standards of mearuement, physiological interpretation, and clinical use. Eur Heart J. 1996;17: 354–381. 8737210

[12] Gerritsen J, Dekker JM, TenVoorde BJ, Kostense PJ, Heine RJ, Bouter LM, et al. Impaired Autonomic Function Is Associated With Increased Mortality, Especially in Subjects With Diabetes, Hypertension, or a History of Cardiovascular Disease: The Hoorn Study. Diabetes Care. 2001;24: 1793–1798. doi: 10.2337/diacare.24.10.1793 11574444

[13] Kleiger RE, Miller JP, Bigger JT, Moss AJ. Decreased heart rate variability and its association with increased mortality after acute myocardial infarction. Am J Cardiol. 1987;59: 256–262. doi: 10.1016/0002-9149(87)90795-8 3812275

[14] Tsuji H, Larson Martin G., Venditti Ferdinand J., Manders Emily S., Evans Jane C., Feldman Charles L., et al. Impact of Reduced Heart Rate Variability on Risk for Cardiac Events. Circulation. 1996;94: 2850–2855. doi: 10.1161/01.CIR.94.11.2850 8941112

[15] Michael S, Graham KS, Davis GM. Cardiac Autonomic Responses during Exercise and Post-exercise Recovery Using Heart Rate Variability and Systolic Time Intervals—A Review. Front Physiol. 2017;8. doi: 10.3389/fphys.2017.00301 28611675 PMC5447093

[16] Shaffer F, McCraty R, Zerr CL. A healthy heart is not a metronome: an integrative review of the heart’s anatomy and heart rate variability. Front Psychol. 2014;5. doi: 10.3389/fpsyg.2014.01040 25324790 PMC4179748

[17] Shaffer F, Ginsberg JP. An Overview of Heart Rate Variability Metrics and Norms. Front Public Health. 2017;5. doi: 10.3389/fpubh.2017.00258 29034226 PMC5624990

[18] Tavakolian K. Systolic Time Intervals and New Measurement Methods. Cardiovasc Eng Technol. 2016;7: 118–125. doi: 10.1007/s13239-016-0262-1 27048269

[19] Ahmed S, Levinson GE, Schwartz CJ, Ettinger PO. Systolic time intervals as measures of the contractile state of the left ventricular myocardium in man. Circulation. 1972;46: 559–571. doi: 10.1161/01.cir.46.3.559 4561119

[20] Cacioppo JT, Berntson GG, Binkley PF, Quigley KS, Uchino BN, Fieldstone A. Autonomic cardiac control. II. Noninvasive indices and basal response as revealed by autonomic blockades. Psychophysiology. 1994;31: 586–598. doi: 10.1111/j.1469-8986.1994.tb02351.x 7846219

[21] Hubert HB, Feinleib M, McNamara PM, Castelli WP. Obesity as an independent risk factor for cardiovascular disease: a 26-year follow-up of participants in the Framingham Heart Study. Circulation. 1983;67: 968–977. doi: 10.1161/01.cir.67.5.968 6219830

[22] Koh-Banerjee P, Wang Y, Hu FB, Spiegelman D, Willett WC, Rimm EB. Changes in Body Weight and Body Fat Distribution as Risk Factors for Clinical Diabetes in US Men. Am J Epidemiol. 2004;159: 1150–1159. doi: 10.1093/aje/kwh167 15191932

[23] Landsberg L, Aronne LJ, Beilin LJ, Burke V, Igel LI, Lloyd‐Jones D, et al. Obesity-Related Hypertension: Pathogenesis, Cardiovascular Risk, and Treatment. J Clin Hypertens. 2013;15: 14–33. doi: 10.1111/jch.12049 23282121 PMC8108268

[24] Farah BQ, Prado WL do, Tenório TR dos S, Ritti-Dias RM. Heart rate variability and its relationship with central and general obesity in obese normotensive adolescents. Einstein São Paulo. 2013;11: 285–290. doi: 10.1590/s1679-45082013000300005 24136753 PMC4878585

[25] Poirier P, Hernandez TL, Weil KM, Shepard TJ, Eckel RH. Impact of Diet-Induced Weight Loss on the Cardiac Autonomic Nervous System in Severe Obesity. Obes Res. 2003;11: 1040–1047. doi: 10.1038/oby.2003.143 12972673

[26] Windham BG, Fumagalli S, Ble A, Sollers JJ, Thayer JF, Najjar SS, et al. The Relationship between Heart Rate Variability and Adiposity Differs for Central and Overall Adiposity. J Obes. 2012;2012: 1–8. doi: 10.1155/2012/149516 22649714 PMC3357556

[27] The Atherosclerosis Risk in Communities (ARIC) Study: design and objectives. The ARIC investigators. Am J Epidemiol. 1989;129: 687–702. 2646917

[28] ARIC Manual 2 Home and Field Center Procedures ARIC Visit 5 and NCS Study Protocol. 2013. Available: https://aric.cscc.unc.edu/aric9/sites/default/files/public/visitdocuments/v5/Manual%202%20Home%20and%20Field%20Center%20Procedures.pdf.

[29] Schroeder EB, Liao D, Chambless LE, Prineas RJ, Evans GW, Heiss G. Hypertension, Blood Pressure, and Heart Rate Variability: The Atherosclerosis Risk in Communities (ARIC) Study. Hypertension. 2003;42: 1106–1111. doi: 10.1161/01.HYP.0000100444.71069.73 14581296

[30] Nunan D, Sandercock GRH, Brodie DA. A Quantitative Systematic Review of Normal Values for Short-Term Heart Rate Variability in Healthy Adults: REVIEW OF SHORT-TERM HRV VALUES. Pacing Clin Electrophysiol. 2010;33: 1407–1417. doi: 10.1111/j.1540-8159.2010.02841.x 20663071

[31] Siedlecka J, Siedlecki P, Bortkiewicz A. Impedance cardiography–Old method, new opportunities. Part I. Clinical applications. Int J Occup Med Environ Health. 2015 [cited 10 Jul 2019]. doi: 10.13075/ijomeh.1896.00451 26159944

[32] Seravalle G, Grassi G. Sympathetic Nervous System, Hypertension, Obesity and Metabolic Syndrome. High Blood Press Cardiovasc Prev. 2016;23: 175–179. doi: 10.1007/s40292-016-0137-4 26942609

[33] Cheung BMY, Li C. Diabetes and Hypertension: Is There a Common Metabolic Pathway? Curr Atheroscler Rep. 2012;14: 160–166. doi: 10.1007/s11883-012-0227-2 22281657 PMC3314178

[34] Hu L, Bentler PM. Cutoff criteria for fit indexes in covariance structure analysis: Conventional criteria versus new alternatives. Struct Equ Model Multidiscip J. 1999;6: 1–55. doi: 10.1080/10705519909540118

[35] Geovanini GR, Vasques ER, De Oliveira Alvim R, Mill JG, Andreão RV, Vasques BK, et al. Age and Sex Differences in Heart Rate Variability and Vagal Specific Patterns–Baependi Heart Study. Glob Heart. 2020;15: 71. doi: 10.5334/gh.873 33150136 PMC7583712

[36] Balasubramanian P, Hall D, Subramanian M. Sympathetic nervous system as a target for aging and obesity-related cardiovascular diseases. GeroScience. 2018;41: 13–24. doi: 10.1007/s11357-018-0048-5 30519806 PMC6423215

[37] Monahan KD. Effect of aging on baroreflex function in humans. Am J Physiol-Regul Integr Comp Physiol. 2007;293: R3–R12. doi: 10.1152/ajpregu.00031.2007 17442786

[38] Esler M, Lambert G, Schlaich M, Dixon J, Sari CI, Lambert E. Obesity Paradox in Hypertension: Is This Because Sympathetic Activation in Obesity-Hypertension Takes a Benign Form? Hypertension. 2018;71: 22–33. doi: 10.1161/HYPERTENSIONAHA.117.09790 29158358

[39] Dimitropoulos G. Cardiac autonomic neuropathy in patients with diabetes mellitus. World J Diabetes. 2014;5: 17. doi: 10.4239/wjd.v5.i1.17 24567799 PMC3932425

[40] Guarino D, Nannipieri M, Iervasi G, Taddei S, Bruno RM. The Role of the Autonomic Nervous System in the Pathophysiology of Obesity. Front Physiol. 2017;8. doi: 10.3389/fphys.2017.00665 28966594 PMC5606212

[41] Licht CMM, Vreeburg SA, van Reedt Dortland AKB, Giltay EJ, Hoogendijk WJG, DeRijk RH, et al. Increased Sympathetic and Decreased Parasympathetic Activity Rather Than Changes in Hypothalamic-Pituitary-Adrenal Axis Activity Is Associated with Metabolic Abnormalities. J Clin Endocrinol Metab. 2010;95: 2458–2466. doi: 10.1210/jc.2009-2801 20237163

[42] von Lewinski D, Bruns S, Walther S, Kögler H, Pieske B. Insulin Causes [Ca ^2+^ ] _i_ -Dependent and [Ca ^2+^ ] _i_ -Independent Positive Inotropic Effects in Failing Human Myocardium. Circulation. 2005;111: 2588–2595. doi: 10.1161/CIRCULATIONAHA.104.497461 15883206

[43] Perpiñan G, Severeyn E, Wong S, Altuve M. Cardiac autonomic modulation in response to a glucose stimulus. Med Biol Eng Comput. 2019;57: 667–676. doi: 10.1007/s11517-018-1913-1 30349959

[44] Sykes CA, Wright AD, Malins JM, Pentecost BL. Changes in systolic time intervals during treatment of diabetes mellitus. Heart. 1977;39: 255–259. doi: 10.1136/hrt.39.3.255 849385 PMC483229

[45] Synowski SJ, Kop WJ, Warwick ZS, Waldstein SR. Effects of glucose ingestion on autonomic and cardiovascular measures during rest and mental challenge. J Psychosom Res. 2013;74: 149–154. doi: 10.1016/j.jpsychores.2012.10.008 23332530

[46] Benichou T, Pereira B, Mermillod M, Tauveron I, Pfabigan D, Maqdasy S, et al. Heart rate variability in type 2 diabetes mellitus: A systematic review and meta–analysis. Malik RA, editor. <PLOS ONE. 2018;13: e0195166. doi: 10.1371/journal.pone.0195166 29608603 PMC5880391

[47] Boer-Martins L, Figueiredo VN, Demacq C, Martins LC, Consolin-Colombo F, Figueiredo MJ, et al. Relationship of autonomic imbalance and circadian disruption with obesity and type 2 diabetes in resistant hypertensive patients. Cardiovasc Diabetol. 2011;10: 24. doi: 10.1186/1475-2840-10-24 21426540 PMC3072316

[48] Jaiswal M, Urbina EM, Wadwa RP, Talton JW, D’Agostino RB, Hamman RF, et al. Reduced Heart Rate Variability Among Youth With Type 1 Diabetes. Diabetes Care. 2013;36: 157–162. doi: 10.2337/dc12-0463 22961570 PMC3526238

[49] Dimova R, Tankova T, Kirilov G, Chakarova N, Grozeva G, Dakovska L. Endothelial and Autonomic Dysfunction at Early Stages of Glucose Intolerance and in Metabolic Syndrome. Horm Metab Res. 2020;52: 39–48. doi: 10.1055/a-0972-1302 31529423

[50] Lopes HF, Corrêa-Giannella ML, Consolim-Colombo FM, Egan BM. Visceral adiposity syndrome. Diabetol Metab Syndr. 2016;8: 40. doi: 10.1186/s13098-016-0156-2 27437032 PMC4950710

[51] Williams SM, Eleftheriadou A, Alam U, Cuthbertson DJ, Wilding JPH. Cardiac Autonomic Neuropathy in Obesity, the Metabolic Syndrome and Prediabetes: A Narrative Review. Diabetes Ther. 2019;10: 1995–2021. doi: 10.1007/s13300-019-00693-0 31552598 PMC6848658

[52] Ross R, Neeland IJ, Yamashita S, Shai I, Seidell J, Magni P, et al. Waist circumference as a vital sign in clinical practice: a Consensus Statement from the IAS and ICCR Working Group on Visceral Obesity. Nat Rev Endocrinol. 2020;16: 177–189. doi: 10.1038/s41574-019-0310-7 32020062 PMC7027970

[53] Ibrahim MM. Subcutaneous and visceral adipose tissue: structural and functional differences. Obes Rev. 2010;11: 11–18. doi: 10.1111/j.1467-789X.2009.00623.x 19656312

[54] Poliakova N, Després J-P, Bergeron J, Alméras N, Tremblay A, Poirier P. Influence of obesity indices, metabolic parameters and age on cardiac autonomic function in abdominally obese men. Metabolism. 2012;61: 1270–1279. doi: 10.1016/j.metabol.2012.02.006 22444779

[55] Koenig J, Windham BG, Ferrucci L, Sonntag D, Fischer JE, Thayer JF, et al. Association strength of three adiposity measures with autonomic nervous system function in apparently healthy employees. J Nutr Health Aging. 2015;19: 879–882. doi: 10.1007/s12603-015-0508-x 26482688 PMC6121712

[56] Koji Y, Tomiyama H, Ichihashi H, Nagae T, Tanaka N, Takazawa K, et al. Comparison of ankle-brachial pressure index and pulse wave velocity as markers of the presence of coronary artery disease in subjects with a high risk of atherosclerotic cardiovascular disease. Am J Cardiol. 2004;94: 868–872. doi: 10.1016/j.amjcard.2004.06.020 15464667

[57] Tomiyama H, Matsumoto C, Shiina K, Yamashina A. Brachial-Ankle PWV: Current Status and Future Directions as a Useful Marker in the Management of Cardiovascular Disease and/or Cardiovascular Risk Factors. J Atheroscler Thromb. 2016;23: 128–146. doi: 10.5551/jat.32979 26558401

[58] Ato D, Sawayama T. Factors associated with high brachial–ankle pulse wave velocity in non-hypertensive and appropriately treated hypertensive patients with atherosclerotic risk factors. Vasc Health Risk Manag. 2017;13: 383–392. doi: 10.2147/VHRM.S144923 29066906 PMC5644576

[59] Su H-M, Lin T-H, Hsu P-C, Chu C-Y, Lee W-H, Chen S-C, et al. A Comparison between Brachial and Echocardiographic Systolic Time Intervals. Hosoda T, editor. PLoS ONE. 2013;8: e55840. doi: 10.1371/journal.pone.0055840 23409059 PMC3567004

[60] Voss A, Schroeder R, Heitmann A, Peters A, Perz S. Short-Term Heart Rate Variability—Influence of Gender and Age in Healthy Subjects. PLoS ONE. 2015;10: e0118308. doi: 10.1371/journal.pone.0118308 25822720 PMC4378923

[61] Chen LY, Zmora R, Duval S, Chow LS, Lloyd-Jones DM, Schreiner PJ. Cardiorespiratory Fitness, Adiposity, and Heart Rate Variability: The CARDIA Study. Med Sci Sports Exerc. 2018; 1. doi: 10.1249/MSS.0000000000001796 30277902 PMC6377325

